# Functional Recovery as a Survivorship Endpoint in Early-Stage NSCLC

**DOI:** 10.3390/cancers18121958

**Published:** 2026-06-16

**Authors:** Giovanni Leuzzi, Filippo Lococo, Beatrice Cosentino, Federica Sabia, Michele Ferrari, Alessandro Pardolesi, Alessia Stanzi, Jury Brandolini, Luigi Rolli, Matteo Calderoni, Clarissa Uslenghi, Piergiorgio Solli

**Affiliations:** 1 Division of Thoracic Surgery, Fondazione IRCCS Istituto Nazionale dei Tumori, 20133 Milan, Italy; beatrice.cosentino@istitutotumori.mi.it (B.C.); federica.sabia@istitutotumori.mi.it (F.S.); michele.ferrari@istitutotumori.mi.it (M.F.); alessandro.pardolesi@istitutotumori.mi.it (A.P.); alessia.stanzi@istitutotumori.mi.it (A.S.); jury.brandolini@istitutotumori.mi.it (J.B.); luigi.rolli@istitutotumori.mi.it (L.R.); matteo.calderoni@istitutotumori.mi.it (M.C.); clarissa.uslenghi@istitutotumori.mi.it (C.U.); piergiorgio.solli@istitutotumori.mi.it (P.S.); 2 Unit of Thoracic Surgery, Catholic University of the Sacred Heart, 00168 Rome, Italy; filippo.lococo@policlinicogemelli.it; 3 Thoracic Surgery Unit, A. Gemelli University Hospital Foundation IRCCS, 00168 Rome, Italy

**Keywords:** early-stage NSCLC, functional recovery, quality of life, survivorship, patient-reported outcomes, allostatic load

## Abstract

Advances in screening, surgical techniques, perioperative care, and multimodality treatment have progressively expanded the population of long-term survivors with early-stage non-small cell lung cancer (NSCLC). However, disease-free survival does not necessarily correspond to complete functional recovery after curative-intent treatment. Many patients continue to experience persistent fatigue, dyspnea, reduced physical activity, impaired exercise tolerance, muscle loss, and deterioration in health-related quality of life despite adequate oncologic control. This narrative review discusses functional recovery as a survivorship endpoint in early-stage NSCLC, focusing on recovery trajectories, physiologic vulnerability, frailty, sarcopenia, rehabilitation, symptom burden, and emerging biologic frameworks such as allostatic load. Increasing evidence suggests that survivorship after NSCLC should not be interpreted exclusively according to recurrence or survival metrics, but also according to the ability to recover physiologic reserve, autonomy, and daily functioning after treatment. Functional recovery appears heterogeneous and influenced by multiple interacting factors, including baseline reserve, systemic inflammation, physical inactivity, behavioral adaptation, and cumulative stress burden. Rehabilitation strategies, structured symptom surveillance, and patient-reported outcomes may help identify vulnerable patients and improve long-term survivorship trajectories. Future survivorship models should probably integrate oncologic outcomes with longitudinal functional assessment to better characterize recovery patterns after treatment.

## Simple Summary

Patients with early-stage non-small cell lung cancer (NSCLC) are increasingly achieving long-term survival after surgery and curative-intent treatment. However, remaining disease-free does not necessarily mean having fully recovered. Many patients continue to experience persistent fatigue, dyspnea, reduced physical activity, muscle loss, impaired exercise tolerance, and deterioration in quality of life long after treatment completion. Despite this, postoperative follow-up is still largely focused on recurrence detection and survival surveillance. This narrative review discusses functional recovery as a survivorship endpoint in early-stage NSCLC. The available evidence suggests that recovery after treatment is heterogeneous and influenced by multiple interacting factors, including physiologic reserve, frailty, sarcopenia, systemic inflammation, symptom burden, behavioral adaptation, and cumulative stress. Rehabilitation, physical activity, nutritional support, and patient-reported outcomes may help identify vulnerable patients and improve long-term recovery. A survivorship-oriented model extending beyond oncologic control alone may better reflect how patients actually recover, adapt, and function after curative-intent treatment.

## 1. Introduction

Over the last two decades, the increasing implementation of lung cancer screening, earlier diagnosis, minimally invasive surgery (MIS), stereotactic radiotherapy, and multimodality treatment strategies has progressively expanded the population of long-term survivors with early-stage non-small cell lung cancer (NSCLC). In parallel, postoperative outcomes have substantially improved, and many patients currently experience prolonged disease control after curative-intent treatment. However, oncologic outcomes alone may not fully reflect the actual survivorship experience of these patients.

Follow-up strategies after curative-intent treatment are still largely focused on recurrence detection and survival surveillance. In clinical practice, patients are commonly considered to be successfully treated if they remain recurrence-free during follow-up. However, even in the absence of recurrent disease, many long-term survivors continue to experience persistent dyspnea, fatigue, reduced physical activity, impaired exercise tolerance, and deterioration in health-related quality of life (HRQoL). In this setting, standardized instruments such as the EORTC QLQ-C30 and the lung cancer-specific QLQ-LC13 have provided a common language for measuring global health status, physical function, and symptoms across different NSCLC trials and survivorship studies [1,2].

Previous studies have shown that respiratory symptoms, fatigue, and functional impairment may persist long after treatment completion in NSCLC survivors [3,4]. In this context, disease-free survival (DFS) does not necessarily imply physiologic recovery.

Importantly, patient-reported outcomes (PROs) and HRQoL measures may also carry prognostic information beyond conventional oncologic variables [5,6,7]. Tumor stage and performance status, in other words, do not tell the entire story. Indeed, the concept of “cure” should be complemented by the ability to maintain an acceptable and sustainable quality of life over time, minimizing long-term toxicities, functional impairment, and symptom burden.

In this review, “functional recovery” (FR) refers to the ability to regain or preserve meaningful physiologic reserve, symptom control, physical activity, autonomy, and daily functioning after curative-intent treatment, independently of oncologic status. Specifically, FR should not be considered synonymous with HRQoL, performance status, or DFS, but rather as the dynamic interaction between physiologic reserve, symptoms, physical functioning, and long-term autonomy after treatment. Despite this growing evidence, FR remains insufficiently characterized in thoracic oncology.

Recovery is often considered a short-term post-op event, whereas clinical experience suggests a heterogeneous process. It is well-known that some patients rapidly regain their pre-treatment functional status, while others remain disease-free but develop persistent physiologic impairment and symptom-driven limitation over time. Such heterogeneity probably reflects different interactions among surgical stress, baseline physiologic reserve, aging, frailty, sarcopenia, and behavioral adjustment after treatment.

Thus, survivorship after early-stage NSCLC should not be interpreted solely in terms of recurrence or standard survival outcomes. In many patients, the real issue starts after treatment completion. FR may represent one of the most clinically relevant yet underrecognized survivorship endpoints after curative-intent treatment.

Unlike conventional HRQoL-focused reviews, the present narrative review does not primarily focus on HRQoL assessment itself, but rather on functional recovery as a multidimensional survivorship endpoint after curative-intent treatment for early-stage NSCLC.

## 2. Search Strategy

This review was developed through a focused review of the literature on survivorship, FR, physiologic vulnerability, symptom burden, rehabilitation, and PROs after curative-intent treatment for early-stage NSCLC. Literature research was primarily conducted using the PubMed database.

The following Medical Subject Headings (MeSH) terms and keywords were used in different combinations: “non-small cell lung cancer”, “early-stage NSCLC”, “survivorship”, “functional recovery”, “quality of life”, “patient-reported outcomes”, “fatigue”, “dyspnea”, “frailty”, “sarcopenia”, “rehabilitation”, “prehabilitation”, “exercise capacity”, and “allostatic load”.

Priority was given to clinically relevant studies, longitudinal analyses, survivorship-focused investigations, and papers exploring recovery trajectories and functional outcomes from a thoracic surgical oncology perspective. Both the original articles and selected review papers published in English were considered. Reference lists of relevant articles were also manually selected to identify additional studies of interest.

Study selection was driven by clinical relevance and by the ability of individual studies to contribute to the conceptual framework of functional survivorship after curative-intent treatment for early-stage NSCLC. Given the narrative nature of this review, a formal systematic review methodology and PRISMA-based study selection process were not applied.

### 2.1. Beyond Recurrence: Why Survival Alone Is Insufficient

The growing number of long-term survivors in early-stage NSCLC has shifted attention toward standard survivorship-related outcomes. While postoperative surveillance mainly focuses on oncologic control, clinical experience suggests that recovery after treatment is more complex than a simple disease-free condition.

Several studies have shown that functional impairment may persist long after treatment completion, even among recurrence-free survivors. Sarna et al. evaluated 142 disease-free NSCLC survivors at least 5 years from treatment. Dyspnea was present in 39% of survivors, wheezing in 31%, phlegm in 28%, and cough in 25%. Notably, 21% of patients reported spending most of the day in bed during the previous year because of respiratory symptoms. Mean forced expiratory volume in one second (FEV1) was 68% predicted, while 36% of survivors had moderate-to-severe obstructive or restrictive ventilatory impairment. Symptom burden appeared more strongly associated with impaired HRQoL than pulmonary function impairment alone [3].

These results indicate that DFS and physiologic recovery may represent partly distinct dimensions of survivorship. From a clinical perspective, many patients remain oncologically cured, while simultaneously experiencing fatigue, exercise limitation, inactivity, and symptom-driven reduction in autonomy. Similarly, Koczywas et al. reported progressive deterioration in symptom burden, physical functioning, and HRQoL in early-stage NSCLC, supporting the concept that survivorship patterns may remain unstable after treatment end [4].

Fatigue is probably one of the most underestimated dimensions of survivorship after lung cancer treatment. In a multicenter study including patients on active treatment and cancer survivors, Wang et al. reported moderate-to-severe fatigue in 45% of patients receiving active therapy and in 29% of survivors. Among survivors, poor performance status was strongly associated with clinically significant fatigue (OR 3.48), as was previous depression history (OR 2.21) [8]. In the Fatigue Coalition study, Curt et al. reported that 76% of patients experienced fatigue at least several days per month during treatment, while 30% reported daily fatigue. More importantly, 91% stated that fatigue prevented them from living a “normal” life, and 75% of employed patients modified their work status [9]. These data show that fatigue is not simply a subjective symptom, but a relevant functional limitation.

Persistent symptoms also tend to interact through multidimensional patterns. Molassiotis et al., in a longitudinal study including 504 symptom assessments during the first year after diagnosis, identified six major symptom clusters that remained relatively stable over time. Patients with symptom clusters had symptom frequency, severity, and distress levels up to 75% higher than the overall cohort [10]. Fatigue, dyspnea, emotional distress, inactivity, and nutritional impairment may therefore represent interconnected manifestations of impaired recovery rather than isolated postoperative complaints.

Functional deterioration may also be objectively measurable. A prospective observational study conducted by Granger et al. on NSCLC patients from diagnosis to 6 months reported lower physical activity levels, reduced quadriceps strength, poorer nutritional status, and impaired HRQoL compared with healthy controls. Approximately 60% of patients did not reach recommended physical activity levels, and functional decline continued during follow-up, with worsening 6-min walking distance, muscle strength, and symptom burden [11].

Similarly, exercise capacity trajectories after lung resection also appear heterogeneous. Brunelli et al. prospectively collected 200 patients undergoing major lung resection and reported significant postop decline in FEV1, diffusing capacity of the lung for carbon monoxide (DLCO), and peak oxygen consumption (VO2 peak). After lobectomy, FEV1 recovered to 84% of baseline values at 3 months, whereas after pneumonectomy it recovered only to 66% of baseline values. Interestingly, some patients with Chronic Obstructive Pulmonary Disease (COPD) had physiologic improvement after lobectomy (probably reflecting a volume-reduction effect in selected hyperinflated lungs), suggesting that baseline reserve and disease distribution may influence recovery patterns [12].

Functional impairment may also carry prognostic information. As reported previously, Quinten et al. showed that baseline physical functioning independently predicted OS in a pooled EORTC analysis from 30 randomized trials (HR 0.94, 95% CI 0.92–0.96; *p* < 0.0001). Pain and appetite loss also had prognostic significance, and adding HRQoL parameters improved prognostic discrimination, with the C-statistic increasing from 0.68 to 0.72, corresponding to an approximately 6% improvement in predictive accuracy [5]. In parallel, Handforth et al., in a systematic review including almost 3000 older cancer patients, reported a median frailty prevalence of 42%. Frailty was associated with increased 5-year mortality (adjusted HR 1.87, 95% CI 1.36–2.57), postop complications (HR 3.19, 95% CI 1.68–6.04), and treatment intolerance (OR 4.86, 95% CI 2.19–10.78) [13].

Particularly in the setting of early-stage NSCLC, these data indicate a significant discrepancy between oncologic control and recovery patterns. Surgical (and multimodal) treatments frequently help to achieve prolonged OS. However, despite these favorable outcomes, some patients rapidly return to good physical activity and social functioning, while others develop progressive exercise limitation and functional decline. Thus, survivorship should not be interpreted as a distinction between recurrence and cure, but rather as a dynamic process characterized by heterogeneous recovery trajectories after treatment.

### 2.2. Functional Recovery Trajectories After Treatment

As already reported before, not all patients recover the same way, and the reasons are rarely reducible to surgical technique or tumor biology only. Many patients regain pre-treatment activity in a few weeks and move on. A relevant subset, however, never returns to baseline, and they adapt, reduce expectations, and stop mentioning it at follow-up visits. Finally, in others, exercise tolerance worsens, fatigue becomes daily, muscle mass quietly disappears, and autonomy contracts. None of this shows up on CT or PET scans.

The available literature suggests that early postoperative decline is expected after lung resection, but the degree and timing of recovery are variable. Physiologic recovery after major lung resection follows domain-specific kinetics: FEV1, DLCO, and VO2 peak do not recover at the same rate, and postoperative deficits may persist across multiple physiologic domains [12]. These data show that postoperative recovery is not uniform across physiologic domains. Lung function, diffusion capacity, and exercise capacity do not necessarily recover with the same kinetics.

The extent of parenchymal resection may also influence postoperative recovery trajectories. Recent randomized evidence has reshaped the surgical management of selected patients with small peripheral early-stage NSCLC. In JCOG0802/WJOG4607L, segmentectomy showed non-inferior relapse-free survival and superior overall survival compared with lobectomy, although the difference in postoperative FEV1 reduction was statistically significant but below the predefined threshold for clinical relevance [14]. Similarly, CALGB 140503 showed that sublobar resection was non-inferior to lobectomy for disease-free survival, with similar overall survival and only a modest preservation of pulmonary function at 6 months [15]. These data suggest that parenchymal preservation may contribute to functional recovery, particularly in selected patients, but they also reinforce the idea that recovery trajectories are not determined by resection extent alone. Baseline physiologic reserve, frailty, comorbidities, and symptom burden probably remain major modifiers of long-term functional survivorship.

PROs may describe a partly different dimension of the same process. Handy et al. reported that patients undergoing lobectomy had reduced HRQoL compared with the general population, even before surgery. At 1 month, the physical composite score decreased from 51 to 45.1 (*p* < 0.0001), but recovered to 52.4 at 3 months, while the mental composite score remained substantially unchanged. Correlations between HRQoL measures and physiologic parameters such as FEV1, DLCO, or stair-climbing performance remained weak overall [16]. Physiologic tests are essential, but they cannot fully replace patient-reported measures when evaluating recovery.

Pompili et al. specifically evaluated predictors of postoperative decline in HRQoL after major lung resections and reported that 28% and 15% of patients experienced clinically relevant deterioration in physical and mental HRQoL components, respectively, at 3 months after surgery [17]. Lower pulmonary reserve and specific baseline HRQoL domains were associated with a higher risk of postoperative decline. The relevant question is not only whether the patient survives surgery, but whether he or she returns to a meaningful functional baseline.

In this setting, baseline physiologic reserve strongly influences recovery patterns. The ACCP guidelines already incorporate these data for preoperative risk assessment. Specifically, patients with predicted postoperative FEV1 and DLCO above 60% are generally considered low risk for anatomical resection, whereas values between 30% and 60% require additional low-technology exercise testing. A stair-climbing height above 22 m or shuttle-walk distance above 400 m identifies lower-risk patients, while VO2 peak below 10 mL/kg/min or 35% predicted indicates high risk for mortality and/or long-term disability after major anatomical resection [18]. These thresholds were designed for operative risk stratification, but they also underline a wider concept: pre-treatment functional reserve is one of the main determinants of post-treatment recovery.

Recovery is also influenced by systemic and muscular factors, not only by lung volume loss. Jones et al. described exercise intolerance in cancer as the result of alterations along the oxygen transport and utilization pathway, including cardiopulmonary, muscular, metabolic, and behavioral components [19]. This concept is highly relevant in early-stage NSCLC. A technically successful resection may still be followed by prolonged inactivity, loss of muscle strength, reduced exercise capacity, and persistent symptoms. As reported by Granger et al., many patients with NSCLC are physically inactive at diagnosis, and functional decline may continue during the first months after treatment [11].

**Figure 1:**
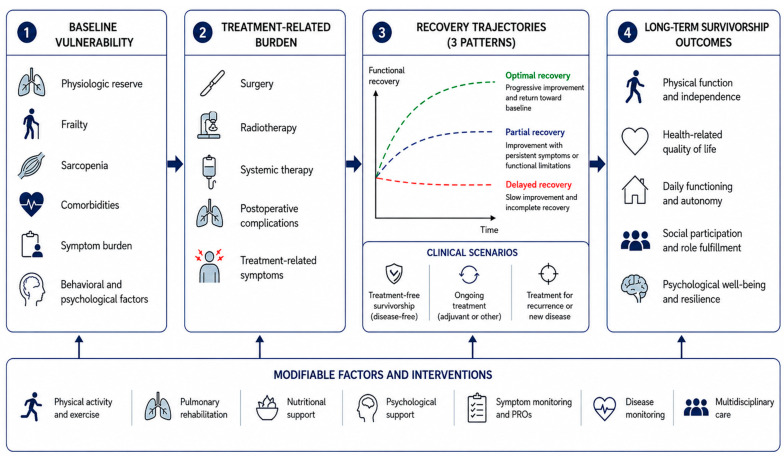
Conceptual framework of functional recovery after curative-intent treatment for early-stage NSCLC.

For these reasons, FR should be evaluated dynamically in both the preoperative and surveillance settings. In fact, a single postoperative assessment may miss significant information, such as delayed recovery, progressive impairment, or secondary decline. In clinical practice, the most useful model should be “trajectory-based”: clinicians should evaluate baseline reserve, early treatment-related decline, partial or complete recovery, and long-term functional stability. This approach may help in identifying distinct patient phenotypes: rapid recovery, slow recovery, persistent impairment, or progressive vulnerability (Figure 1). These data may be more clinically informative than isolated postoperative values.

FR trajectories may become an important endpoint of survival after curative-intent treatment for early-stage NSCLC. They link surgical stress, baseline reserve, symptoms, physical activity, and long-term autonomy into a single clinically meaningful framework. More importantly, they shift the focus from “what was removed” or “whether recurrence occurred” to “how the patient actually recovers after treatment”.

### 2.3. Physiologic Vulnerability: Frailty, Sarcopenia, and Reduced Functional Reserve

One of the main limitations of traditional oncologic outcomes is that they insufficiently capture baseline physiologic vulnerability. Patients with apparently similar tumor stage, pulmonary function, and performance status may experience profoundly different patterns of recovery after curative-intent treatment. Frailty, sarcopenia, nutritional status, and reduced functional reserve represent some of the most important determinants of functional survivorship after treatment. The main determinants of impaired FR are summarized in Table 1.

Frailty should not simply be interpreted as advanced age. Fried et al. originally defined frailty as a biologic syndrome defined by reduced reserve and diminished resistance to stressors, associated with weakness, slowed performance, low physical activity, weight loss, and exhaustion [39]. Later, Clegg et al. further conceptualized frailty as a state of elevated vulnerability resulting from age-associated decline across multiple physiologic systems, leading to impaired homeostasis after stressful events [40]. This concept is particularly relevant in thoracic oncology, where surgery, systemic inflammation, hospitalization, inactivity, and postop complications may all contribute to destabilizing already vulnerable patients.

Several studies have shown that frailty significantly affects postoperative and long-term outcomes after cancer treatment. As previously reported in a systematic review with almost 3000 older cancer patients, Handforth et al. documented a median frailty prevalence of 42%, and with frailty associated with increased mortality, postoperative complications and treatment intolerance [13]. Ethun et al. also stressed the relevance of frailty across oncologic surgical populations, where it may identify patients at risk of major postoperative complications and non-home discharge independent of age alone [20]. Recent thoracic and lung-cancer-focused studies have additionally reinforced frailty as a clinically relevant marker of prognosis and perioperative vulnerability [21]. This aspect is clinically relevant because many patients considered technically operable may, on the other hand, be frail and have limited physiologic reserve. Performance status alone often fails to capture this frailty, particularly in older patients who seem independent but poorly tolerate physiologic stress. In these patients, postoperative decline may reflect limited recovery capacity as much as treatment-related injury.

In this heterogeneous scenario, sarcopenia is one of the main biological manifestations of this vulnerability. Progressive loss of skeletal muscle mass and muscle function has consistently been associated with worse postoperative outcomes, reduced tolerance to stress and oncologic therapies, and impaired survival across multiple cancer settings. Prado et al. contributed to establishing the clinical concept of “sarcopenic obesity” in oncology, highlighting its association with adverse treatment-related outcomes [22]. Subsequently, Martin et al. analyzed a large cohort of more than 1400 patients with respiratory and gastrointestinal cancers and showed that low muscularity was strongly associated with shorter survival independently of body mass index (BMI) [23]. These findings changed the interpretation of body composition in oncology, showing that BMI alone may inadequately reflect physiologic reserve. In thoracic oncology, sarcopenia is particularly relevant because respiratory function, exercise tolerance, and postoperative recovery are closely dependent on muscular performance. Shachar et al. confirmed in a systematic review and meta-analysis that CT-defined sarcopenia was associated with significantly worse OS across different solid tumors [24]. Similar observations have been reported specifically in NSCLC populations where decreased skeletal muscle mass correlated with increased postoperative morbidity, poorer physical functioning, and shorter survival [25].

In addition, sarcopenia should not be interpreted only as a radiologic finding. Muscle loss frequently coexists with inactivity, fatigue, systemic inflammation, nutritional impairment, and reduced exercise capacity. In this setting, it may represent both a marker and a mediator of impaired FR. As reported before, Granger et al. showed that many NSCLC patients already present low physical activity levels and reduced muscle performance at diagnosis [11]. Treatment-related inactivity may further amplify this process, leading to progressive deconditioning and reduction in physiologic reserve over time.

Cancer cachexia represents the end of this context. Baracos et al. described cancer-associated cachexia as a complex systemic syndrome involving muscle wasting, metabolic dysfunction, inflammation, anorexia, and reduced functional capacity [30]. Fearon et al. defined cancer cachexia as a multifactorial syndrome characterized by ongoing skeletal muscle loss that cannot be fully reversed by conventional nutritional support and progressively leads to functional impairment [31]. Although cachexia is more frequently associated with advanced disease, early NSCLC patients may develop milder forms of muscle wasting and nutritional decline during the perioperative phase. As a result, European Society for Clinical Nutrition and Metabolism (ESPEN) guidelines further support systematic nutritional assessment and intervention in cancer patients, strengthening the need to include nutrition within survivorship-oriented care pathways [32].

### 2.4. Can Functional Recovery Be Modified?

If FR can be defined as a trajectory rather than as a single event, functional decline need not be considered inevitable. Prehabilitation, postoperative rehabilitation, nutritional support, symptom observation, and exercise-based programs may all contribute to modifying long-term recovery.

Exercise and rehabilitation have been extensively evaluated in lung cancer populations, although with heterogeneous designs, endpoints, and timing. In a Cochrane review on preoperative exercise training in NSCLC, Granger et al. highlighted that prehabilitation may improve exercise capacity before surgery, although evidence remained limited by small sample size and heterogeneity across studies [33]. Similarly, a meta-analysis performed by Sebio Garcia et al. reported that preoperative exercise training was associated with improved functional capacity and postop outcomes in patients undergoing lung cancer surgery [41]. In practical terms, these studies suggest that at least part of physiologic reserve may still be modifiable before surgery.

Randomized studies also suggest that short-term pulmonary rehabilitation may have measurable perioperative benefits. Benzo et al. evaluated preoperative pulmonary rehabilitation before NSCLC resection in two randomized studies and reported reductions in median hospital stay (4 vs. 6 days) and chest tube duration (4 vs. 8 days) after surgery [42]. Morano et al. conducted a pilot randomized trial comparing preoperative pulmonary rehabilitation with chest physical therapy in patients undergoing lung cancer resection and observed improvements in exercise capacity and postoperative outcomes [43]. Lai et al. similarly showed that short-term pulmonary rehabilitation before lobectomy improved perioperative functional parameters and postoperative recovery [34]. Although these studies were relatively small, they remain clinically relevant because they suggest that FR may still be modifiable even within the short time window before surgery.

Postoperative exercise is also important. A Cochrane review of exercise training within 12 months after lung resection for NSCLC performed by Cavalheri and Granger reported that exercise interventions improved functional exercise capacity after surgery, with pooled improvements in 6-min walk distance exceeding 40 m in some analyses [35]. More recently, Voorn et al. reviewed the effects of prehabilitation and rehabilitation on HRQoL and fatigue in NSCLC patients undergoing surgery, confirming that exercise-based strategies were consistently associated with improvements in physical functioning and fatigue-related outcomes [44]. These results are particularly relevant for functional survivorship, as they shift the focus from complication prevention alone to the positive concept of restoration of autonomy, activity, and daily functioning.

Rehabilitation may also improve symptoms and functional independence in more advanced lung cancer settings, supporting the biologic plausibility of intervention across the disease history. In this setting, Henke et al. reported improved Barthel Index scores in the intervention group compared with controls (92.08 vs. 81.67; *p* = 0.041), together with improvements in physical functioning, 6-min walk test, stair walking, muscle strength, and dyspnea during submaximal walking [45]. Although this study was not focused on early-stage postoperative survivorship, it reinforces the concept that fatigue, dyspnea, and reduced independence are potentially modifiable clinical targets.

The implementation of rehabilitation is still a challenge in daily practice. Despite its impact on daily functioning, cancer-related fatigue is still underrecognized and often inadequately discussed during follow-up visits. Berger et al. stated that fatigue may persist for a long time after treatment completion and may interfere with usual activities, supporting the need for systematic screening and structured supportive interventions [46]. Borneman et al., evaluating implementation of fatigue guidelines at a NCCN member institution, identified barriers at the patient, professional, and system levels, including poor documentation, limited referrals, and the expectation that clinicians should initiate the discussion [47]. In thoracic oncology, this gap is arguably worse than in other cancer settings: stage I NSCLC patients are often told they are cured, discharged from oncologic care, and never systematically screened for fatigue or functional decline. HRQoL questionnaires are rarely administered in thoracic surgical outpatient clinics as well, and when they are, they are often regarded as optional rather than standard of care. This has to change.

In this evolving scenario, digital and home-based models may further expand the concept of functional follow-up. Lung cancer survivors have expressed interest in telerehabilitation after curative-intent therapy, particularly when programs are flexible, individualized, and compatible with post-treatment limitations [48]. In parallel, Silver et al. emphasized that cancer rehabilitation may improve function in survivors and reduce the individual and societal burden of cancer-related disability [49]. For early-stage NSCLC, this suggests that follow-up should not only ask whether recurrence has occurred, but also whether the patient is walking, breathing, sleeping, eating, and living as before treatment. Thus, rehab interventions should be largely integrated into survivorship pathways rather than offered only after functional decline has already become established.

### 2.5. Biological Vulnerability: Inflammation, Stress Response and Allostatic Load

Increasing evidence suggests that biologic vulnerability, systemic inflammation, behavioral adjustment, and cumulative physiologic stress may all contribute to the shaping of long-term FR after treatment.

The concept of “allostatic load” may help interpret part of this heterogeneity. McEwen and Stellar originally described allostatic load as the cumulative physiologic burden generated by chronic exposure to stress and repeated activation of adaptive systems [26]. Over time, persistent inflammatory, neuroendocrine, metabolic, and behavioral alterations may progressively impair patient’s resilience and reduce the ability to recover from additional stressors. In thoracic oncology, surgery, hospitalization, pain, inactivity, nutritional impairment, fatigue, sleep disruption, and systemic inflammation may all contribute to this cumulative burden. In early-stage NSCLC, major lung resection may itself act as a relevant physiologic stressor. In some patients, especially those who are frail, sarcopenic, or already deconditioned before surgery, postoperative pain, inactivity, sleep disturbance, and reduced autonomy may contribute to prolonged functional decline despite adequate oncologic control.

Importantly, allostatic load should not be interpreted as simply psychological stress. Mathew et al. showed that elevated allostatic load was associated with worse outcomes among different cancer populations, supporting the concept that cumulative biologic dysregulation may affect both prognosis and survivorship [27]. More recently, Obeng-Gyasi et al. reported that elevated allostatic load was associated with worse OS in metastatic NSCLC patients, further supporting its possible clinical relevance in lung cancer populations [28]. Although studies specifically focused on early-stage survivorship remain limited, the biologic plausibility of this model is strong. At present, the allostatic load is not routinely measured in thoracic oncology practice, and standardized perioperative applications in early-stage NSCLC are still lacking. This concept is particularly relevant after treatment, when persistent symptoms, reduced physical activity, muscle loss, fatigue, and chronic low-grade inflammation frequently coexist and progressively interact over time. In this context, allostatic load may help explain why some patients develop persistent functional vulnerability despite adequate oncologic control.

In the setting of allostatic load, systemic inflammation probably represents one of the most relevant biologic links between cancer, treatment-related stress, and persistent functional impairment. Bower and Lamkin illustrated the relationship between inflammation and cancer-related fatigue, suggesting that inflammatory activation may contribute to persistent symptoms even beyond active treatment phases [50]. Roxburgh and McMillan showed that markers of systemic inflammatory response are strongly associated with survival across several solid tumors [51]. In early-stage NSCLC, persistent inflammatory activation may therefore reflect not only oncologic aggressiveness, but also impaired physiologic recovery capacity, reduced resilience, and progressive functional vulnerability after treatment.

Recent studies have started to directly connect allostatic load with PROs and long-term recovery. Chen et al. reported an association between elevated allostatic load and shorter time to deterioration of HRQoL in NSCLC patients [52]. Ishibe et al. also discussed the potential role of allostatic load as an epidemiologic measure to integrate stress exposure, cancer risk, progression, and mortality [29]. Although this field is still evolving, these findings support the idea that biologic vulnerability and patient-reported recovery may represent closely interconnected dimensions rather than separate survivorship domains.

Behavioral factors can add to this process. Smoking exposure, physical inactivity, social isolation, poor nutrition, and impaired mobility may all contribute to inflammatory and metabolic imbalances over time. Guan et al. recently reported interactions between allostatic load, smoking exposure and lung cancer risk, supporting this larger concept that cumulative physiologic stress may contribute to both disease development and post-treatment vulnerability [53].

These concepts have direct clinical consequences. Actually, postop follow-up built solely around recurrence surveillance is not sufficient for NSCLC patients. Careful evaluation of symptoms, nutritional status, physical activity, and identification of vulnerable patients should become part of our routine during surveillance. From a clinical perspective, allostatic load may help integrate inflammation, body composition, functional measures, and behavioral factors when identifying patients at risk of prolonged functional impairment. For early-stage NSCLC survivors, it may provide more insight into where a patient ends up 6 months after surgery than the pathology report ever will.

### 2.6. Clinical Implications and Future Directions

Serial PROs may help address part of this gap. As reported before, HRQoL and symptom burden may carry prognostic information beyond conventional clinicopathologic variables [5,6,7]. More importantly, repeated symptom assessment may identify patients drifting toward progressive functional impairment before decline becomes clinically evident. Velikova et al. showed that systematic symptom measurement improved patient-physician communication and patient well-being [36]. Similarly, Basch et al. showed that structured PRO monitoring during routine cancer treatment improved symptom control, HRQoL, emergency room access, and treatment tolerance [37]. Subsequent analyses from the same trial also suggested improved OS among patients under systematic electronic symptom surveillance [38]. These outcomes are relevant because they suggest that longitudinal symptom assessment may not only influence HRQoL, but also clinically meaningful oncologic outcomes. However, it should be acknowledged that most of these data were generated in patients undergoing active cancer treatment, frequently in advanced disease settings, and their direct applicability to long-term surveillance after curative-intent treatment remains to be established.

In thoracic surgical oncology, functional decline after curative-intent treatment is usually gradual. Patients may gradually reduce physical activity, social interaction, and exercise tolerance while still appearing clinically stable during standard oncologic follow-up. Longitudinal symptom assessment may thus become more informative than isolated postoperative evaluations.

Digital models may help facilitate this evolution. Denis et al. performed a randomized trial comparing web-mediated follow-up with routine surveillance in lung cancer patients, and showed that remote symptom monitoring may improve clinical management and facilitate earlier identification of postop relevant events [54]. More recently, Jovanoski et al., reviewing real-world evidence in early-stage NSCLC survivors, highlighted the growing relevance of survivorship-oriented outcomes and the persistent gap between oncologic control and patient-perceived recovery [55].

Although they are not difficult to administer, implementing survivorship-oriented models actually remains challenging. The 2026 NCCN guidelines for cancer-related fatigue recommend systematic screening and management of fatigue during and after cancer treatment: particular attention should be given to functional status, deconditioning, physical activity, sleep disturbance, nutritional deficits, and multidisciplinary supportive care pathways [56]. This recommendation fits particularly well with early-stage NSCLC survivorship, where persistent fatigue and reduced physical activity are often underappreciated during postoperative follow-up.

As treatment paradigms continue to evolve, survivorship models should also account for the growing use of perioperative systemic therapies in resectable NSCLC. Immune checkpoint inhibitors and targeted therapies have introduced new patterns of chronic toxicity, including fatigue, endocrine dysfunction, musculoskeletal symptoms, and pulmonary adverse events, which may contribute to cumulative physiologic burden beyond surgery alone. Although these approaches have substantially improved oncologic outcomes in selected patients, their long-term implications for functional recovery remain incompletely characterized and warrant further investigation within survivorship-oriented frameworks [57].

Another important implication is related to survivorship phenotyping. Future models would need to integrate physiologic reserve, frailty, body composition, inflammatory burden, physical activity, allostatic load, and PROs into integrated recovery profiles rather than relying exclusively on recurrence-based definitions.

This perspective may also influence perioperative decision-making itself. Functional vulnerability may become increasingly relevant not only for estimating perioperative risk, but also for anticipating long-term functional outcomes after treatment. The ability to recover may eventually become as clinically relevant as the ability to tolerate treatment.

Overall, survivorship after early-stage NSCLC is progressively evolving from a purely oncologic concept toward tailored models combining tumor control, physiologic resilience, symptom burden, and long-term functional recovery. The next challenge will not be limited to prolonging survival alone, but rather to understanding how patients actually live after treatment and which aspects of recovery can still be modified over time.

In addition, ethical considerations are also central to this paradigm shift: when survival benefits are increasingly achievable, clinicians and researchers have a responsibility not only to prolong life but also to ensure that survivorship remains meaningful, dignified, and sustainable for patients. This requires balancing treatment efficacy with long-term toxicity, promoting shared decision-making, and recognizing the patient’s perspective as a fundamental endpoint of care.

### 2.7. Limitations of the Review

This review has several limitations. First, it was designed as a narrative review and therefore does not follow a formal systematic-review methodology. Second, the literature search was restricted to PubMed, potentially excluding relevant studies indexed in other databases. Third, evidence directly linking allostatic load to functional recovery in early-stage NSCLC remains limited and largely indirect. Finally, some of the evidence supporting longitudinal symptom monitoring and PRO-based interventions derives from patients undergoing active treatment, frequently in advanced disease settings, and its applicability to long-term surveillance after curative-intent treatment should therefore be interpreted with caution.

## 3. Conclusions

Disease-free is not the same as being well. This distinction, obvious to anyone who follows these patients over time, is still largely absent from how we design postoperative follow-up after curative intent treatment for early-stage NSCLC. Contemporary oncologic strategies have substantially improved disease control and long-term OS, but a recurrence-free status alone does not necessarily correspond to complete physiologic or FR. Many patients continue to experience persistent fatigue, dyspnea, inactivity, reduced exercise tolerance, muscle loss, and impaired HRQoL long after treatment completion. Survivorship should therefore not be interpreted as a binary condition, defined exclusively by the absence of recurrence.

The available evidence suggests that recovery after treatment follows different trajectories influenced by baseline physiologic reserve, frailty, sarcopenia, systemic inflammation, behavioral adaptation, and symptom burden. This perspective also has important clinical implications. Post-treatment follow-up may need to evolve beyond radiologic surveillance alone to a wider survivorship-oriented model including symptom assessment, physical activity, rehabilitation approaches, nutritional evaluation, and PROs. In parallel, increasing evidence suggests that several dimensions of functional decline may be at least partially modifiable through pre-habilitation, rehabilitation, structured symptom surveillance, and assisting interventions.

The concept of functional survivorship does not aim to replace conventional oncologic outcomes, but rather to complement them. Long-term survivorship after early-stage NSCLC should be evaluated not only according to how long patients live after treatment, but also according to how they recover, adapt, and function over time.

In practical terms, FR may emerge as one of the most clinically relevant survivorship endpoints in early-stage NSCLC. Subsequent research will need to better define recovery phenotypes, identify patients at risk of persistent impairment, and develop integrated survivorship models that combine oncologic control with preservation of long-term function and autonomy. This will also require more systematic integration of HRQoL, PROs, pulmonary function, and exercise capacity into both clinical trials and routine postoperative care. In the coming years, the challenge will not simply be to increase the number of survivors after early-stage NSCLC, but to understand which patients truly recover after treatment and which remain “trapped” in persistent functional vulnerability despite disease control.

## Figures and Tables

**Table 1:** Main determinants of impaired functional recovery after curative-intent treatment for early-stage NSCLC.

Domain	Clinical Implications for Functional Recovery	References
***Frailty***	Reduced physiologic reserve, impaired stress tolerance, increased postoperative complications and prolonged recovery	Handforth et al. [13]; Ethun et al. [20]; Hu et al. [21];
***Sarcopenia***	Muscle loss, reduced exercise capacity, impaired postoperative recovery, worse long-term outcomes	Prado et al. [22]; Martin et al. [23]; Shachar et al. [24]; Suzuki et al. [25]
***Symptom burden***	Persistent fatigue, dyspnea, inactivity, and reduced autonomy despite disease control	Sarna et al. [3]; Wang et al. [8]; Curt et al. [9]; Molassiotis et al. [10]
***Reduced exercise capacity***	Lower physical activity, progressive deconditioning, impaired functional independence	Granger et al. [11]; Brunelli et al. [12]; Handy et al. [16]; Jones et al. [19]
***Systemic inflammation and allostatic load***	Persistent biologic vulnerability, impaired resilience, chronic functional decline	McEwen and Stellar [26]; Mathew et al. [27]; Obeng-Gyasi et al. [28]; Ishibe et al. [29]
***Nutritional impairment and cachexia***	Reduced muscle function, impaired recovery, progressive physiologic decline	Baracos et al. [30]; Fearon et al. [31]; ESPEN guidelines [32]
***Rehabilitation and physical activity***	Potentially modifiable determinants of recovery and long-term autonomy	Granger et al. [32]; Sebio Garcia et al. [33]; Cavalheri and Granger [34]; Voorn et al. [35]
***Patient-reported outcomes (PROs)***	Early identification of vulnerable patients and longitudinal monitoring of recovery trajectories	Quinten et al. [5]; Scott et al. [6]; Velikova et al. [36]; Basch et al. [37,38]

## Data Availability

No new data were created or analyzed in this study.
